# An Enactive-Ecological Approach to Information and Uncertainty

**DOI:** 10.3389/fpsyg.2020.00588

**Published:** 2020-04-21

**Authors:** Eros Moreira de Carvalho, Giovanni Rolla

**Affiliations:** ^1^Department of Philosophy, Institute of Philosophy and Human Sciences, Federal University of Rio Grande do Sul, Porto Alegre, Brazil; ^2^Department of Philosophy, Faculty of Philosophy and Human Sciences, Federal University of Bahia, Salvador, Brazil

**Keywords:** Shannon-information, information for action, information as covariance, enactivism, ecological psychology, uncertainty

## Abstract

Information is a central notion for cognitive sciences and neurosciences, but there is no agreement on what it means for a cognitive system to acquire information about its surroundings. In this paper, we approximate three influential views on information: the one at play in ecological psychology, which is sometimes called information for action; the notion of information as covariance as developed by some enactivists, and the idea of information as a minimization of uncertainty as presented by Shannon. Our main thesis is that information for action can be construed as covariant information, and that learning to perceive covariant information is a matter of minimizing uncertainty through skilled performance. We argue that the agent’s cognitive system conveys information for acting in an environment by minimizing uncertainty about how to achieve intended goals in that environment. We conclude by reviewing empirical findings that support our view by showing how direct learning, seen as an instance of ecological rationality at work, is how mere possibilities for action are turned into embodied know-how. Finally, we indicate the affinity between direct learning and sense-making activity.

## The Quarrel About Information

Information is the bread and butter of cognitive science and neuroscience (CSN). Talk about information *processing*, *control*, *storage*, and *retrieval* is abundant in explanations of how cognitive systems can perform specific tasks and enable agents to interact intelligently with their environment. Accordingly, one of the defining tasks of CSN is to describe the mechanisms through which information is conveyed, an enterprise that, if successful, allows us to understand, predict, simulate, and intervene upon the cognitive capacities of real agents.

The groundwork of the way information is understood by CSN today was laid by Shannon’s (1948) mathematical account of information, which made possible nothing less than digital communication. Simply put, Shannon’s theory defines information as entropy, which is the measure of average uncertainty of the selection of an encoded signal. The core idea of what became known as *Shannon-information* is that the less uncertain the selection of the encoded signal is at its receiver, the more information the signal carries from its sender. Noise, on the other hand, permanently corrupts the signal, thus increasing entropy and diminishing information. To summarize, information is a matter of minimization of uncertainty. Thus, CSN requires the cognitive system to fundamentally receive and process encoded signals in a way that minimizes uncertainty about their source, say, the distal causes that initiate cognitive processing^1^.

It might seem straightforward that the converse of Shannon-information is *representational content*—despite Shannon’s advertence that “[the] semantic aspects of communication are irrelevant to the engineering problem” (Shannon, 1948, p. 379). Indeed, proponents of CSN typically—but not necessarily, or so we will argue—take the encoded signals processed by cognitive systems to carry information *about* their source, which implies that the signals display semantic content, i.e., they (non-metaphorically) *tell* the cognitive system something about its environment^2^. Homunculus issues aside—if the signal tells something to the cognitive system, then something within the system is listening after all—a dominant idea behind CSN is that the outcome of information processing is the composition, manipulation, and consumption of representational content. Patterns of neural activity, therefore, supposedly *represent* whatever gave rise to the cognitive acts of which they are a token, because they convey information about their distal sources.

Two parallel research programs in cognitive sciences and psychology, however, challenge the theoretical tenability of the very notion of representational content, or at least the assumption according to which representational content is needed to explain all sorts of cognitive activity. On the one hand, Gibson’s (2015) ecological psychology gave rise to a research program that identifies environmental variables, which are known as *affordances*, as directly (non-representationally) perceived by cognitive agents. According to the ecological view, agents directly perceive possibilities of engagement with their immediate environment according to their specific bodily morphologies. On the other hand, the general outlines of what would later become known as enactivism were first presented by Maturana and Varela (1980) and later expanded by Varela et al. (1991). Enactivists argue that cognition is not a matter of representing the environment, it is instead the active exploration of an environment by an organism, which determines meaningful points of interest for an organism with specific systemic structures—an activity that is known as “sense-making.”

The consolidation of both paradigms characterized the so-called *Pragmatic Turn* in the cognitive sciences (Engel et al., 2013) and, given the shared rejection of pervasive representations in cognition and semantic notions more generally, pragmatically oriented views of cognition typically eschew traditional informational parlance. Gibson straightforwardly rejected that Shannon-information could serve as perceptual information (Gibson, 2015, pp. 231–232), given the communicative character of information in Shannon’s view. Perceptual information, for Gibson, is not communicative because it is direct, it cannot be a matter of *translating* the messages emitted from a source. A more critical stance toward semantic information has recently been developed by radical enactivists (Hutto and Myin, 2013). They claim that natural structures, such as patterns of neural activity, do not exhibit accuracy conditions, which is the defining trait of representational content—whatever else representations turn out to be. What they call the Hard Problem of Content is the challenge of reducing structures with accuracy conditions to the physical world. Given that the promise of reduction has not been fulfilled, so their argument goes, the assumption that representational content is pervasive of all cognition, turns out to be a matter of theoretical recklessness. They write:

“Anything that deserves to be called content has special properties—e.g., truth, reference, implication—that make it logically distinct from, and not reducible to, mere covariance relations holding between states of affair (Hutto and Myin, 2013”, p. 67).

Covariance is the relation of two or more states of affairs varying reliably or nomically, and if that relation holds, it does not follow that one state of affairs represents the other. It is one thing to say that smoke *indicates* fire, meaning that whenever there is smoke there is fire; it is another thing altogether to say that smoke *says* that there is fire, or *stands for* fire. And it is the former that grounds a scientifically respectful notion of information, one that evades the Hard Problem of Content. Hutto and Myin call it *information as covariance*. So, “if information is nothing but covariance then it is not any kind of content—at least, it is not content defined, even in part, in terms of truth-bearing properties” (Hutto and Myin, 2013, p. 67). Importantly, if two variables co-vary reliably, one can *predict* the value of a variable based on the value of the other (Anderson and Chemero, 2013). This kind of consideration casts a different light on what a deflationary notion of information may look like within the enactive paradigm: cognitive systems co-vary reliably with their environment, in a way that an external observer can observe, for instance, patterns of brain activity and predict their source, given the correct set of assumptions regarding the broader mechanisms that play a role in cognitive activity (bodily morphology and environmental display, for instance). According to this less contentious notion of information, cognitive systems are, to use a Gibsonian metaphor, “attuned” to their medium.

Interestingly, criticism from the radically enactive camp is not directed solely to cognitivism, which is the philosophy underpinning the dominant view on CSN. They argue that Ecological Psychology and what they call Autopoietic Enactivism—Maturana and Varela’s original ideas regarding sense-making and autopoiesis—end up smuggling representational content through semantic information. Despite Gibson’s emphatic rejection of representationalism and Shannon-information (at least for perception), his recurrent phrase “information pickup” puts its view under suspicion of covert representationalism: “no informational content is ‘picked up’ or ‘extracted from’ the world and then ‘supplied’ to the user by sensory means” (Hutto and Myin, 2013, p. 73). For, if there is no informational content, so they might argue, there is nothing to be picked up by the cognitive system. More recently, discussing Tony Chemero’s (2009) account, which combines ecological psychology with dynamic systems theory, Hutto and Myin write that:

“Chemero’s version of an ecological dynamical approach […] remains committed to the language, if not the framework, of information processing. Some of Chemero’s ways of talking—when he speaks of the “pro-vision,” “use,” “gathering,” and “pickup” of information “about” affordances—are anathema to a non-representational rendering of Gibson (Hutto and Myin, 2017”, p. 86).

But as van Dijk et al. (2015) argue, and Hutto and Myin acknowledge (Hutto and Myin, 2017, pp. 86–87), this does not necessarily put radical enactivism at odds with ecological psychology. The key to reconcile both paradigms is to take the Gibsonian notion of information not as carrying content *about* the medium, but as offering possibilities of action *for* an agent, something that becomes clearer if we take Gibson’s notion of affordance seriously, as we will do in the following section. Following van Dijk, Withagen, and Bongers, we call this view *information for action*. Moreover, as Segundo-Ortin et al. (2019) argue, *specification* and *meaning*, which are central notions for ecological psychology, are compatible with the principles of radical enactivism. To say that the information in the ambient array specifies the environment amounts to saying that there is a lawful covariation between patterns of the array and the environment—a point we also stress below. Accordingly, the notion of *meaning* is also free from contentful worries in ecological psychology because information is “meaningful” for the organism in the sense that it is acquired through active exploration of its environment, which is a goal-directed activity.

In this paper, we side with pragmatic views of cognition, for we reject pervasive representational content and semantical information as the basis of all cognition. But we part ways on the supposed relation between information as a minimization of uncertainty and representational content—Shannon’s idea was precisely that a quantitative account of information is independent of semantic issues. Thus, we offer an account of information as a minimization of uncertainty without representational content. In section “Ecological Information (or Information *for* Action)” we turn to the notion of information in ecological psychology in order to provide more details of a pragmatically oriented account of information, that is, information for action. We also show how this account of information offers an interesting opportunity to approximate ecological psychology to enactivism. Finally, we indicate that perceptual learning is a process of minimization of uncertainty, a point we will further develop in the last section based on the empirical literature. In section “Skilled Agency and Information” we intend, on the one hand, to capture the idea of reduction of uncertainty that underlies Shannon-information, but without implying representational content and, on the other hand, to be consistent with the idea of information for action. We argue that the agent’s cognitive system conveys information for acting in an environment by minimizing uncertainty about how to achieve the intended goals in that environment through skilled agency. This idea is compatible with enactivism for, as we show, can be cast in terms of information as covariance. We address the challenge of explaining how skilled agency, which is refined and reinforced through past interactions, can be adapted to deal with unforeseen circumstances and successfully minimize uncertainty in new cases. We conclude in section “Direct Learning and Minimization of Uncertainty” by reviewing empirical findings that support our view and by showing how direct learning, seen as an instance of ecological rationality at work, is the core engine by which mere possibilities for action are turned into embodied know-how. Finally, we indicate the affinity between direct learning and sense-making activity.

## Ecological Information (Or Information *For* Action)

The notion of ecological information is central to understanding perception and perceptual learning within the ecological approach to perception. This kind of information is non-representational and non-sensorial at the same time, opening up a unique path to ecological psychology in the study of perception that does not resemble traditional empiricist or cognitivist approaches. In order to capture the core features of ecological information, it will be helpful to contrast it with sensory stimulation.

The concept of stimulus is used in different ways in psychology and physiology, and even within psychology there is no agreement about how it should be defined precisely (Gibson, 1960). For instance, “does a stimulus motivate the individual,” considered from the first-personal perspective, “or does it merely trigger a response” (Gibson, 1960, p. 695), which could happen only at the subpersonal level? One may also wonder whether a stimulus necessitates a behavioral response or not. Finally, there has also been debate about whether a stimulus activates a sense organ or not, in other words, whether it is effective or just potential (Gibson, 1960, p. 696). It seems that all depends on how far we want to go into the environment to explain changes—physiological, behavioral, or dispositional—in the organism. In perceptual science, it is common to assume that a stimulus is a form of physical energy—optical, acoustical, mechanical, or chemical—that, by exceeding a certain threshold, effectively activates a receptor (Gibson, 2015, p. 46). Sensory stimulation is then that passive process of receptor activation. The *stimulus energy* at issue is proximal, punctate, and momentary, since it is the immediate cause for the activation of a single receptor at a given time (Gibson, 1960, p. 698).

Empiricist and cognitivist approaches to perception share the assumption that sensory stimulation provides the starting point for the study of perception. They differ, however, in how they conceive perception. For the empiricist, perception boils down to the sensations that follow sensory stimulation and their associations, whereas, for the cognitivist, perception is about objects and events in our three-dimensional environment, it produces perceptual states that represent the distal causes of sensory stimulation, as it is typically done in CSN. As the stimulus energy carries no information about the environment (Gibson, 1960, p. 699), it needs to be processed and enriched for the construction of these perceptual representations (Gibson, 2015, p. 240).

Gibson rejects the assumption above, thereby, rejecting both the empiricist and cognitivist views of perception. In its place, he puts forward the view that, going deeper into the environment, we can find distal, structured, and persisting potential stimulus, which he calls *stimulus information* (Gibson, 1968, p. 29; Gibson, 2015, p. 47). The first thing to notice is that energy can be ordered and structured over time and/or space. Differences of intensity may form a pattern in these two dimensions. For instance, a point of observation in ambient light has structure if the light at that point is different in different directions (Gibson, 2015, p. 45). Structure matters because it can specify the environment, in particular its source. In the case of ambient light, its “structure is locally predictable; that is, physics could, in principle, provide a point by point accounting of reflection and absorption” (Michaels and Carello, 1981, pp. 21–22). Thus, the structure of ambient light specifies surfaces and their properties in that the former is lawfully related to the latter. In a similar way, ambient light structured over time may specify patterns of change, namely, events. Information, in the ecological approach to perception, is just that relation of specification (Gibson, 1960, p. 702; Gibson, 1968, p. 245; Gibson, 2015, p. 231). Accordingly, stimulus information is structured energy to which an organism may be sensitive. Before we characterize in more detail how the organism becomes sensitive to stimulus information, some clarifications are in order.

We mentioned in the last section that Gibson’s talk about information and, as we will soon discuss, the process of picking up information have raised concerns, mainly from radical enactivists (Hutto and Myin, 2017, p. 122), as to whether ecological psychology is radical enough and really uncommitted to representations. We think that these concerns are unfounded. The relation of specification upon which ecological information rests is nothing more than nomic covariation (Gibson, 1968, p. 244; Heras-Escribano, 2019, p. 150), which is a respectable naturalistic notion according to radical enactivists themselves (Hutto and Myin, 2013, p. 71; Hutto and Myin, 2017, p. 67). For Gibson, it is absolutely clear that ecological information is devoid of semantic or contentful information: “The connection between natural stimuli and their sources is not the same as the connection between social stimuli and their sources, for example, the connection between words and their referents. This latter problem, surely, is distinct. Semantics is one thing, ecology is another” (Gibson, 1960, pp. 699–700).

Ecological information is not present in any kind of covariation. First, a structure that specifies its source must be causally related to that source, in that changes in the source are followed by changes in the structure. Accidental or casual covariation is unsafe for grounding organism’s perceptions and actions. Second, that relation of specification might be local, that is, the structure might specify its source only under certain conditions or, more precisely, in the organism’s niche. For instance, a bioelectric field that is “partially modulated in the rhythm of the living thing’s respiratory movements” (Turvey et al., 1981, p. 276) specifies an edible thing in the environment where sharks live, for “in the niche of the shark ‘an edible thing’ and ‘electric field of, say, type F’ are nomically related” (Turvey et al., 1981, p. 277). Thus, local covariation may be enough for specification. Finally, there has been a debate among ecological psychologists about whether the covariation must be strong enough to support a 1:1 specifying relationship or just a probabilistic specifying relationship (Heras-Escribano and de Pinedo, 2016; Bruineberg et al., 2018). In the latter case the environmental structure does not *uniquely* specify its source because the correlation between them is not exception-free. For instance, the covariation between smoke and fire is less than perfect in that the occurrence of the first makes the occurrence of the latter only likely. Bruineberg, Chemero, and Rietveld distinguish between lawful and general ecological information to capture respectively strict and probabilistic covariation (Bruineberg et al., 2018, pp. 6–7). Of course, the former is just a special case of the latter. What is up for grabs is whether probabilistic covariation is enough to support ecological information. On the one hand, there are plenty of non-strict regularities in the environment, natural or social, that could be useful to guide behavior. Having access to information that some event is likely, is better than having no information whatsoever. On the other hand, general ecological information opens up the possibility of perceptual error. In those occasions in which an environmental structure is present but not its likely source, such as in the case of smoke without fire, an organism may pick up the general optical information about fire when there is no fire around. This would be a case of perceptual error. However, the ecological approach to perception is committed to direct perception, which precludes cases of perceptual error as traditionally conceived (Gibson, 1968, p. 287; Heras-Escribano and de Pinedo, 2016, p. 581; Segundo-Ortin et al., 2019, p. 1016). Cases of misperception in the ecological approach are not cases of picking up information that fails to point to its source but cases of failing to pick up information.

Giving up direct perception is an option, but it would presumably throw us back to empiricist or cognitivist views of perception (Gibson, 2015, p. 159). Besides, it is hard to see how one could account for perceptual error without assuming a suspicious intermediate, maybe representational, between the perceiver and the world. We take a different path. The gap between general and lawful ecological information can be overcome by taking the local aspect of regularities seriously. In general, the occurrence of smoke may indeed make the occurrence of fire only likely, but it may uniquely specify fire in a particular environment. Organisms are not expected to be sensitive to information irrespective of where they find themselves, on the contrary, they might be able to manifest their sensibility to a piece of information only in their niches. As in the example of sharks above, electric field of a certain type locally specifies edible things. As Gibson, and following Raja’s suggestion that a new law-based psychology is Gibson’s most radical idea (Raja, 2019), we reject that cues or mere probable correlations are sufficient to ground and explain perception (Gibson, 1957). To deal with non-universal correlations, we appeal to Runeson’s distinction between complete and incomplete invariants. This is not a distinction between specifying and non-specifying invariants, both kinds of invariant specify some feature of the environment, but the former depends on constraints that hold throughout the relevant environment, whereas the latter depends additionally on further constraints which do not apply throughout the relevant environment (Runeson, 1989, p. 7). Thus, the relevant distinction is not between general and lawful information, but between local and universal lawful information. In this way we preserve ecological information as a 1:1 specifying relationship^3^.

Ecological information has a dual nature, it is not only, as we have been discussing, information *about* the environment but also information *for* the organism. In fact, an environmental structure is information about something only because it is detectable and usable by an organism, but not because it is semantically laden as assumed in traditional CSN. Thus, ecological information is information in relation to an organism, it specifies both the environment and the organism (Gibson, 2015, p. 132). Let us unpack these claims. The organism needs to be considered in the study of information for three reasons. First, information, as a relevant category in behavioral explanations, cannot fulfill its function to point to something unless it is detectable. So, an energy pattern can be information for an organism only if the organism has sensory registers that are sensible to that kind of energy. Ultraviolet radiation, even if structured, is not information for beings like us, but it can be for honeybees. Second, the detection of energy structured over time and/or space depends on the organism’s abilities to explore its environment. Third, and more importantly, for information to be usable it must be detected in a way that is meaningful or intelligible to the organism. According to Gibson, what an organism perceives when it looks at objects is not their physical qualities but their affordances, what the organism can do with them (Gibson, 2015, p. 126). As perception is direct, ecological information must then specify affordances too (Gibson, 2015, p. 131). This result shouldn’t come as a surprise since organisms live not in the environment as such but in a particular niche, “a setting of environmental features that are suitable for an animal” (Gibson, 2015, p. 121). A kind of organism implies a kind of niche and *vice versa*, they are complementary, a niche “complements the variety of actions a species must perform” (Michaels and Carello, 1981, p. 44). Thus, the ecological information specifying those aspects or features of the environment that normally call the organism’s attention also specifies their affordances. When we focus on the affordances specified by the ecological information, information is personal, it is information for a species or for an individual^4^.

Take, for instance, the information for optical contact. This information specifies the time at which an object would collide with an observer. When an object is coming toward the observer, it progressively occupies a wider area of the observer’s visual field until the limit in which it occupies the whole field, the moment of the collision. According to Gibson and a later study by Lee (1976), the observer does not use information about the absolute speed and distance to calculate the time of contact, as a cognitivist would hypothesize. Rather, they directly pick up the rate of optical expansion of the object. This information is enough to guide the observer’s behavior because “the rate of magnification is proportional to the *imminence* of collision” (Gibson, 2015, p. 167). This example is interesting because it shows in a clear way the dual nature of ecological information. The rate of optical expansion of an object specifies a type of event, the *approach-of-something* (Gibson, 2015, p. 167). This is information *about* an event. At the same time, this information is body-scaled, it relates the approaching object to the observer’s visual field. Thus, the rate of expansion is also information *for* an organism inasmuch as it specifies possibilities for action, such as receding, deviating, or preparing for collision. As Michaels and Carello put forward:

“As with the example of approaching, the flow of optical texture specifies what is happening (walking toward) and what is about to happen (imminence of collision). Beyond this, the actor requires that the information be in a usable form. This means that it must be specific to the animal (body-scaled) and specific to the animal’s particular environment. Perceptual information is specific to the event and compatible with the level of regulation involved in activity (Michaels and Carello, 1981”, p. 54).

As said before, there is nothing contentful in the ecological notion of *information about*. We need to keep in mind that, according to the ecological psychology, perception is not a state of the organism considered in isolation from the environment but of the whole organism-environment system (Lombardo, 2017, p. 3; Richardson et al., 2008, p. 170; Raja, 2019, p. 801). An organism perceives only when it is coupled to an environment, living and enacting in its niche. Only those aspects or features of the environment to which the organism is attuned in a practical way, by knowing how to deal with them, constitute the most immediate lived world of the organism, its niche, that region of the environment *about* which the organism can have perceptions. Although we can abstract structured energy from how it is detected by an organism, leaving out what that structured energy affords, the fact is that “for structured energy to qualify as information, an animal not only must have an ability to detect that information, it must also have a way to use it” (Michaels and Carello, 1981, p. 46). Thus, information *for* is the key to information *about*.

Finally, the ecological notion of *information for* and the ecological view of perceptual learning offer an interesting opportunity to approximate ecological psychology to enactivism. Assuming Shannon’s idea of information as minimization of uncertainty and the enactivist view of sense-making as the activity by which an autonomous system regulates its coupling with the environment in an adaptive way (Di Paolo, 2015), ecological psychology can bring both ideas together in its explanation of perceptual learning. For Eleanor Gibson and James Gibson, perceptual learning is a discriminative process by which the organism’s differential responses to ecological information get richer with practice (Gibson and Gibson, 1955, p. 39). Whenever learning is successful, the organism is “in closer touch with the environment” (Gibson and Gibson, 1955, p. 34) in that it becomes attuned to information that specifies affordances of something in the environment. Understood in this way, perceptual learning is also a process of minimization of uncertainty in that the organism moves from a situation in which its environment is undifferentiated, an indefinite number of possibilities for action are on a par with each other, to a situation in which particular affordances show up to the organism. Becoming attuned to information that specifies affordances is how the organism gets away from uncertainty. As Eleanor Gibson points out, “detecting unity, order, and redundancy are all ways of reducing uncertainty and of achieving specificity and economy” (Gibson and Pick, 2000, p. 157)^5^.

## Skilled Agency and Information

The ecological notion of information provides the conceptual link between the idea of minimization of uncertainty, which is central to Shannon-information, and nomic variation or reliable covariation, which is the “scientifically reliable notion” endorsed by radical enactivists. So far, we have shown that, with due adjustments, these different views of information can be made to converge without implying representational content or semantically laden information. What is missing from this picture, however, is the role played by skilled agency in minimizing uncertainty.

Since its early days, enactivists have emphasized the role played by agency in cognition. The initial motivation in Maturana and Varela’s work (Maturana and Varela, 1980) was to explain the distinctiveness of living organisms, with the additional supposition that whatever makes an organism a living one makes it a cognitive agent as well—which later became known as the strong life-mind continuity thesis (de Jesus, 2016), as endorsed for instance by some enactivists like Thompson (2007). Autopoiesis, the continuous production of the organism’s own components and its functional distinction from the outside environment, was thus conceived in order to explain the difference between agency and mere mechanic reaction. An autopoietic organism is constituted by a precarious network of interrelated processes that determine its own viability conditions through self-production, approaching favorable conditions for its existence and avoiding detrimental ones. This, however, is insufficient in explaining agency, given that favorable and detrimental viability conditions can vary in degrees (Di Paolo, 2005). The fuller picture is that a cognitive organism is not only autopoietic but *adaptive*, that is, it improves its viability conditions by selecting more favorable environmental couplings and avoiding more detrimental ones, altering the set of parameters and conditions that affect the dynamic coupling between agent and environment (Di Paolo et al., 2017). We take this modulation of the system-environment coupling to be a matter of conveying information, which can be understood at the personal level as *skilled agency*.

Consider the following scenario: an inexperienced agent finds herself in a situation where she intends to do something. That can be achieved through certain actions that are available to her—however, due to her inexperience, she is uncertain about the outcome of any particular action in that environment. To add more details to that scenario, imagine a child using pointy cutlery for the first time with the intention of eating something on their plate, or a beginner piano student struggling to coordinate their hands while playing a scale. Plausibly, both are cases of intentional action, even though the agents in question may lack the ability to describe their intentions in a fine-grained manner. We recognize, therefore, a goal in their actions, and how well they perform depends on how close they get to achieve their goals. Importantly, their inexperience translates to uncertainty about the outcomes of specific actions *vis-à-vis* their goals, for they have a plethora of ways of acting and no means of choosing the most efficient or least costly ones. That is, unskilled agents cannot discriminate between more and less favorable environmental couplings for the achievement of specific goals. The child may hold the fork and the knife in a way that may be inefficient to cut the meal and bring it to their mouth, whereas there are many alternative ways to hold the fork and the knife which can be more efficient than the way they have done it—and they presently lack the cognitive resources to make a decision for a better way. Similarly, the piano student may play the scale incorrectly or out of sync due to the way they are placing their fingers on the keys, thus failing to perceive that there is a more efficient way within the set of possible ways to play that scale in a piano with a certain weight to its keys, and so on.

What the examples above show is that an inexperienced agent does not perceive the relevant possible ways of acting as clearly and as well-defined as a more experienced agent would. A skilled agent, on the other hand, has established efficient ways of achieving specific goals in those circumstances (and sufficiently similar ones), which means that they are more certain that a specific way of acting has the desired outcome in those circumstances. Thus, skilled agency minimizes uncertainty, conveying more information for action—how someone should act, given those circumstances (and sufficiently similar ones)—for the best way of doing something varies nomically with the intended outcome. Given that an agent’s performance is selected and refined in order to deal with specific circumstances, that is, it is developed through their engagements within their niche, the information conveyed is usually local rather than universal, but in cases of skilled performance it is also uniquely specified in those particular circumstances.

The talk about uncertainty naturally leads to the question of whether we’re committed to an objective interpretation of uncertainty, according to which it is inherently probabilistically unmeasurable, or to a subjective interpretation, which intends to treat objective uncertainty as subjective estimations of specific outcomes for specific actions. The latter option would allow for uncertainty to be treated in the way risk sometimes is in economy and decision theory, that is, as a case in which each action leads to specific outcomes whose probabilities are known by the agent, but which are not certain. Naturally, a more suitable approach to the enactive-ecological view of information is the ecological interpretation of uncertainty put forth by Kozyreva and Hertwig (2019), which was inspired by bounded and ecological rationality (Simon, 1956; Todd and Gigerenzer, 2012). Their view is that uncertainty is a function of the systemic coupling between agent and environment, an emergent feature that depends on how the agent engages with her niche. What Kozyreva and Hertwig call “uncertainty as a property of the organism-environment system” is a needed change to the concept of uncertainty for the enactive-ecological approach of information, given that both enactivism and ecological psychology take the system comprised of *agent interacting in an environment* to be the fundamental unit of analysis. Thus, “uncertainty comprises both environmental unpredictability and uncertainties that stem from the mind’s boundaries, such as limits in available knowledge and cognitive capabilities” (Kozyreva and Hertwig, 2019). Our previous discussion shows that we should include *skillfulness* in the class of “cognitive capabilities” that affect uncertainty, for, the more skillful the agent is, the more information they acquire from their surroundings. Moreover, as Kozyreva and Hertwig acknowledge, their view of uncertainty as an emergent feature of the agent-environment system leads to the idea that, in order to understand how the organism deals with uncertainty, it is crucial to understand their evolved cognitive capacities and the strategies they have developed in order to engage with their environment. Conversely, the way the organism explores the information that is available for them depends not only on their skills, but also on their bodily morphology, both from the ontogenetic and the phylogenetic standpoints. Clearly, bodily morphology selects and restricts the set of possible actions an individual can undertake in order to achieve a certain goal, functioning as the most fundamental factor in the minimization of uncertainty.

Aside from bodily morphology and skill, it should be clear due to our emphasis on intentional action that another variable to factor in the minimization of uncertainty is the practical interest, or simply the goals, of the agent in that environment. That the agent’s goals matter in information pick up is one of the morals to be drawn from Neisser and Becklen (1975) classic ballgame experiment, where subjects watched two superimposed videos of basketball players passing the ball and, given their task of counting the number of passes between players in one video, they typically didn’t notice “odd events”—which included, in replications of that experiment, a lady passing by with an umbrella and the famous gorilla. Experiments of selective attention therefore show that information that is plainly available to the agent is not picked up if it does not affect their goals. Accordingly, individuals with similar bodily morphologies and similar skill levels can still perform widely different actions in the same environment given their goals.

Now, if the skilled agent minimizes uncertainty about the outcomes of their actions, thus having a rich informational pickup going on, due to the limited set of actions they can undertake in order to accomplish a given task; it might look puzzling how the skilled agent is able to deal with unforeseen circumstances. After all, their skillfulness enables them to *limit* the set of possible actions, whereas unforeseen circumstances, at least the ones that don’t relate to more familiar one’s, may as well call for *new* actions. So, paradoxically, it might seem that the skillful agent would be less apt to deal with new circumstances and environments. This is, in fact, plausible: the reliance upon habits, that is, patterns of engagement we reinforce in order to act more skillfully in familiar settings, may set us back when we face new situations. But that is not to say that the unexperienced performer would be at an advantage, for they also would be greatly uncertain of the outcome of their actions in those circumstances. However, we speculate that in such cases the skilled agent would still be in a favorable position, because their skills enable them to operate at a higher order, perceiving the similarities between familiar environments and new ones, thus adapting previously selected pairs of actions/outcome to engage in new, more suitable actions.

## Direct Learning and Minimization of Uncertainty

The literature on direct learning (Jacobs et al., 2000, 2001, 2009; Michaels et al., 2008) helps us to bring together and provide empirical support for some claims we made in the last two sections, namely, that agents perceive by picking up ecological information specific to affordances and that skilled agents minimize uncertainty about the outcomes of their actions. As we pointed out in section “Ecological Information (or Information *for* Action),” energy patterns may correlate with their respective sources in different degrees. However, according to the ecological approach, only a 1:1 specifying relationship supports direct perception. If this assumption about direct perception is correct, one can predict that learning to perceive “involves moving across the information manifold to a locus that permits better performance in the task” (Michaels et al., 2008, p. 944), in other words, through perceptual learning one is expected to change from relying on local specifying variables to universal specifying variables when these are available and more useful to the task at hand. This should not come as a surprise, since skilled performance seems to require successful perception in a variety of circumstances. We will discuss one study which obtained this result, namely, convergence to use universal specifying variables after practice.

The study in question tracked the variable to pick up the relative mass of colliding balls (Jacobs et al., 2009). Based on a prior study (Runeson, 1983), in which it was shown that the kinematics of linear collisions contain unambiguous information about kinetic properties, such as weight ratio, Jacobs et al. proposed a set of experiments to test whether novice and expert observers would differ in the kinematic information they use to perceive the relative mass of colliding balls (Jacobs et al., 2009, p. 1019). At least three types of kinematic information about colliding balls are correlated with their relative masses. As pointed out by Runeson, the mass ratio of colliding balls is specified by the amount of velocity change, according to the following formula: *m*1/*m*2=|*v*1−*u*1|/|*v*2−*u*2|, where *m1* and *m2* are the masses of the balls, *u1* and *u2* are the velocities of the balls before the collision, and *v1* and *v2* are the velocities of the balls after the collision. The amount of velocity change is a very useful variable because it highly correlates with mass ratio across different environments. Another two kinematic variables that *might* be highly correlated with mass ratios are the difference in exit speeds—the speeds of the balls after the impact—and the difference in scatter angles—the angles between a ball’s velocity before and after collision (Jacobs et al., 2001, p. 1019). These are local non-specifying variables in that they highly correlate with mass ratios only in some specific conditions. In Jacobs et al.’s experiments, collisions between balls were simulated by a computer and displayed in a screen to observers who were then instructed to estimate the relative mass of the colliding balls. The experiments were designed to track learning, they consisted of three sets of trials: an initial 64-trial pretest without feedback, followed by two 74-trial blocks of training with feedback—observers were informed about the correct mass ratios of the balls— and a final 64-trial posttest without feedback. By tweaking the simulation, it was possible to set up a set of trials in which mass ratios were highly correlated with all three variables above. Thus, in one experiment they were able to test whether observers would change the variable they rely on if it correlates highly with mass ratios. In this case, they did not, even those who started relying on local variables (Jacobs et al., 2001, p. 1023). In another experiment, with a different set of trials, where only the universal specifying variable correlated highly with mass ratios, the observers did change the variables they used and converged on the universal specifying one (Jacobs et al., 2001, p. 1032). A higher level of skilled performance was also observed in this case, as remarked by the authors: “Those observers who discover a specifying variable improve dramatically and reach high levels of performance” (Jacobs et al., 2001, p. 1033)^6^.

These results back some claims we made in the last two sections up. We said that the distinction between general and lawful information could be overcome by taking into consideration local constraints. As we mentioned in section “Ecological Information (or Information *for* Action),” both local and universal lawful information allow accurate performance in a task ecology^7^. In the first experiment, the observers kept using the same local specifying variable they started with, the exit speeds, because in the simulated condition that variable was highly correlated with mass ratios and, therefore, was very useful for the task at hand. At the same time, as shown by the second experiment, observers converged to a more useful variable when it was available and the variables they started with were poorly correlated with mass ratios. Change of variable happens when the observer is not already attuned to their task. Thus, the general conclusion is that “observers merely search for variables that are useful in the ecology encountered in practice” (Jacobs et al., 2001, p. 1035), what can be achieved by relying on local or universal information insofar as that information, given universal or local constraints, is useful for the task at hand.

Jacobs et al. finish their paper by advising that “great care must be taken in the selection of a stimulus set; otherwise, what may appear to be global cognitive principles can, in fact, be local solutions to local problems” (Jacobs et al., 2001, p. 1035). However, one may draw a different moral from their results, namely and in resonance with our discussion in the last section, that cognition and rationality should be understood ecologically, as bounded by the environment, the task at hand, and the skills of the agent; as Kozyreva and Hertwig point out, “the essence of rational behavior consists in how an organism can adapt to achieve its goals under the constraints of its environment and its own cognitive limitations” (Kozyreva and Hertwig, 2019). Learning a perceptual skill is a process of adaptation to a discriminatory task, ecologically situated, whereby the agent becomes attuned to ecological information and minimizes the uncertainty about how to act in their environment. Skilled action optimizes the agent-environment coupling, which means that specific actions for the skilled agent have less uncertain outcomes. As we incorporate a skill to our network of skills, our body prepares itself for a set of possible states in the neighborhood of the states we are in as we act. We know what the consequences of our actions will be, not because we have an internal model, but because we are sensitive to these consequences insofar as we are prepared for them and know how to deal with them. Thus, to be sensitive to an affordance is to be less uncertain about the consequences of our actions, and this is the minimization of uncertainty provided by learning to perceive ecological information. As we have already pointed out in sections “Ecological Information (or Information *for* Action)” and “Skilled Agency and Information,” the range of possibilities for actions that can be successfully performed to achieve a goal or solve a task is determined and specified through perceptual learning. Uncertainty, as unpredictability due to a lack of knowledge, even probabilistic knowledge (Gigerenzer, 2019), is thus minimized by turning, through practice, some hitherto mere possibilities for action, whose consequences were also unknown, into embodied know-how. The skillful agent who knows how to *ϕ* enacts a world where the consequences of their *ϕ*-ing are under their control and are felt as such.

By approaching direct learning as an instance of ecological rationality at work, we also make it easier to see this process as close to sense-making activity, as we have already indicated in section “Ecological Information (or Information *for* Action).” Both are adaptive processes enacted by an agent. Gibson also characterizes direct learning as a process of *education of attention* (Gibson, 1968, p. 51; Gibson, 2015, p. 235) by which the agent *selects* only information that is needed for accomplishing their goals (Gibson, 1968, p. 286) and whose outcome is the agent getting “in closer touch with the environment” (Gibson and Gibson, 1955, p. 34). This is not so far away from the sense-making activity or “the capacity of an autonomous system to adaptively regulate its operation and its relation to the environment depending on the virtual consequences for its own viability as a form of life” (Di Paolo et al., 2018, p. 33), where adaptivity is understood as an agent’s ability to distinguish and select what is good and what is bad for the preservation of their own identity over time. We submit that enactive approaches could benefit from adopting the framework of ecological information; direct learning can be a helpful way to frame and explain, at least in part, the capacity behind sense-making activity, and at the same time, we acknowledge that ecological psychology can improve its understanding of the organism-environment systems by encompassing the enactivist emphasis on agency (Stapleton, 2016, p. 326) and the role of the asymmetry between organism and environment, which lies on the side of the former, in explaining how an enacted world is brought forth, as we did in section “Skilled Agency and Information.”

Could this enactive-ecological approach to information and uncertainty scale up and explain how information is conveyed in offline cognitive acts, such as planning, remembering, inferring, hypothesis formulation, and language use? The question assumes that all cognitive performances could be explained within the same framework. While this is a possible position, we do not need to commit to it. It could be the case, for instance, that perception shares more similarities with planning and remembering than it does with inferring and language use, and it could be the case that at least some higher cognitive performances are currently better explained by appealing to more traditional views of information processing^8^. We do, however, believe that the discussion above shows that perceiving and planning do have a lot in common: while the former is a matter of online cognition, the latter is usually taken to be an offline performance, whereby the agent does not need to be in direct contact with their environment. But given that skilled performance involves an embodied readiness to deal with the outcome of our actions, it turns out that perceiving is already a matter of being able to engage in and to deal with possibilities that have not yet been actualized through our actions, what Kiverstein and Rietveld (2018) call “sensitivity to virtual conditions”^9^. Thus, planning could in principle be approximated to perception in this framework. Similarly, recent discussions on procedural memory—that is, the ability to remember how to do something—shows its approximation with perceptual abilities (Hutto and Myin, 2017). Moreover, Michaelian and Sant’Anna, 2019 have convincingly argued that a dispositional conception of memory traces, as favored by post-causal theories of memory; entails that episodic memory functions by strengthening the connection among nodes in a network, not by storing content This again shows that episodic memory, and not only procedural memory, can be understood in a similar manner as perception is in the enactive-ecological framework. It follows that contentful information is not as central to the explanation of episodic memory as traditional CSN would have it, and it opens up the possibility of approximating the way information is conveyed in memory to the way it is in perception. It remains to be seen what other *prima facie* offline cognitive performances could be explained in a similar fashion, but we remain cautiously neutral on whether an enactive-ecological approach is sufficient to do so.

## Concluding Remarks

We have argued that information, stripped of any semantic or contentful significance, can also be the bread and butter of the enactive-ecological research program. Shannon-information as minimization of uncertainty is well placed to work out a bridge between ecological psychology and enactivism. First, we have put forward the ecological view of information as a relation of specification based on covariation. Because of its dual nature, ecological information specifies its source and affordances for an organism. Ecological information is mainly *for* action. Then, based on the enactivist view of agency, we explained minimization of uncertainty as resulting from the skillful activity of an agent while pursuing their intended goals in a particular environment. This is compatible with the ecological view of information because, in fact, what the agent is doing is reducing the full range of available affordances to those that are effective for achieving their goals. New and unknown situations offer new opportunities for an agent to minimize uncertainty, which they face with the help of their already acquired skills. We backed this view of minimization of uncertainty up by appealing to empirical literature on direct learning. Agents converge to use more useful and specifying variables, thus minimizing uncertainty, when their perceptual skills are improved by practice. We also indicated how closely related direct learning and sense-making activity are. Finally, we submit that enactivists should welcome ecological talk about information, since such talk enlightens the non-representational transactions between the organism and environment; and ecological psychologists should forget Gibson’s qualms about Shannon’s view of information, as direct learning can be seen as a process of minimization of uncertainty.

## Author Contributions

EC is the main contributor responsible for sections “Ecological Information (or Information *for* Action)” and “Direct Learning and Minimization of Uncertainty,” whereas GR is the main contributor responsible for sections “The Quarrel About Information” and “Skilled Agency and Information.” Both have made contributions to all sections. Both authors contributed to manuscript revision, read, and approved the submitted version.

## Conflict of Interest

The authors declare that the research was conducted in the absence of any commercial or financial relationships that could be construed as a potential conflict of interest.

## Footnotes

1 Shannon-information has become the cornerstone of the Predictive Coding paradigm (Friston, 2009; Clark, 2012, 2013; Hohwy, 2013, 2016), which is in its tracks to become the dominant one in CSN, given its promise of explanatory unity of a wide range of cognitive phenomena.

2 Importantly, Dretske (1981) criticized Shannon’s information theory precisely because it did not account for the semantic content of information in a more general sense. The criticisms from radical enactivism presented below are initially directed against teleosemantic theories of content/information in general, of which Dretske is a prime example—but, given the assumption by some adherents of CSN that encoded signals carry information *about* their source, the same criticisms apply to how information is sometimes conceived in CSN.

3 van Dijk and Kiverstein (2020) recently proposed a different strategy, which they called *usage-based account of information*. They want to give up the idea that specifying information is pre-given in the sense that there are lawful correlations between surfaces and ambient energy prior to the agent’s unfolding activities. Correlations continue to be necessary for perception in the authors’ view, but they are generated by the activity of the agent. Although van Dijk and Kiverstein keep specification as fundamental for information, in agreement with the orthodoxy in ecological psychology, they part ways from this orthodoxy by proposing to think of specification “as a process in which the organism-environment relation forms” (van Dijk and Kiverstein, 2020). Accordingly, “affordances get specified in doing. Specification of affordances is not something lawfully structured energetic arrays can do on their own” (van Dijk and Kiverstein, 2020). Their main point is that generated lawful correlations between surfaces and the ambient energy get their information significance only through the agent’s unfolding activities to maintain the organism-environment fit. In this sense, “specification is how the resulting patterns of light, sound and all the rest, are used” (van Dijk and Kiverstein, 2020). We agree in part with van Dijk and Kiverstein. It is true that some relevant correlations are brought forth only by the agent’s unfolding activities. We also agree on the importance of how correlations are used, in fact, we acknowledge below that *information for* is the key to *information about*. But we disagree that actual use is what turns correlation into informationally significant. Instead, correlations get their information significance because of what they afford to an agent. For more on this difference between actual use and possible use in the characterization of information for, see Segundo-Ortin et al. (2019, pp. 1015–1016). Finally, in thinking of specification as a process and affordances as getting specified along this process, van Dijk and Kiverstein forgo the relevant distinction between the process of perception and the process of learning to perceive. As we argue below, what is ambiguous, uncertain or indeterminate is not the affordance itself. Instead, given that there are always a great number of affordances available, uncertainty is a matter of which one should be selected by the agent. In learning to perceive the agent learns to select which affordance she should attend to in order to achieve her goals, thus minimizing uncertainty about which affordance she should act upon.

4 According to the ecological approach, an energy pattern is meaningful only if it specifies a possibility for action in relation to an organism. It may be interesting to note the congruence of this view with MacKay’s action-oriented definition of meaning: “the meaning of a message can be defined very simply as its selective function on the range of the recipient’s states of conditional readiness for goal-directed activity; so that the meaning of a message to you is its selective function on the range of your states of conditional readiness. Defined in this way, meaning is clearly a relationship between message and recipient rather than a unique property of the message alone” (MacKay, 1969, p. 24), where *message* is to be understood physically, as patterns of energy (MacKay, 1969, p. 20). As in ecological psychology, it is not actual behavior or usage that confers meaning to an energy pattern but a possibility for action, or, in MacKay’s terms, a conditional readiness for goal-directed activity.

5 Perceptual learning yields a change in the organism-environment system (Szokolszky et al., 2019, p. 11), at the end of the process the organism and its environment are more coupled to each other than before. The higher the specificity achieved, the lower the uncertainty about which affordances are appropriate to the task at hand, and less effort and exploratory activities are necessary for the organism to satisfy its needs. In sum, “over learning and development, there is a continual increase in predictability and efficiency of perceiving what is doable” (Adolph and Kretch, 2015, p. 130).

6 For another very interesting study with similar results, see Michaels et al. (2008). In this study the aim is to track variables for perceiving the length of unseen rods through dynamic touch (Gibson, 1962). For instance, when wielding and hefting a rod one may become attuned to variables that correlate with rod length such as the first and the third principal moments of inertia, or a higher order variable that is a combination of the first two (Michaels et al., 2008, p. 946). As in Jacobs et al.’s study, Michaels et al. (2008, p. 952) also concluded that “perceptual learning is guided by convergence information.”

7 These considerations about the role of local and universal constraints also handle the objection that progressive perceptual learning seems to be incompatible with direct perception, which is a matter of all or nothing. At each stage of learning, the agent is attuned to a different variable, changing from local to more universal ones inasmuch as becoming more skillful in dealing with a task requires accurate performance in a wider range of circumstances. Perceptual learning is a matter of discovering more useful information (Runeson, 1983, p. 8). See also Michaels et al. (2008, pp. 946–947) discussion on information space.

8 Alternatively, Di Paolo et al. (2018) offer a distinct unified explanation of cognition, which consists not in scaling up basic levels of cognition, but instead in *scaling down* what is usually called higher-cognition. As De Jaegher points out in another paper, “nothing in enactive theory restricts it to this so-called ‘low level”’ (De Jaegher, 2019) in that the very understanding of higher-order cognitive phenomena is transformed when it is reconceived by enactive resources. For instance, a correct understanding of embodiment allows us to see how our linguistic bodies and language emerge from a certain kind of participatory sense-making activity without having to appeal to semantic concepts (Di Paolo et al., 2018, p. 215).

9 Although they do subscribe to a strict continuity between lower and higher cognition, a thesis we remain neutral about
